# Emergências Relacionadas à Doença Valvar Cardíaca: Uma Revisão Abrangente da Abordagem Inicial no Departamento de Emergência

**DOI:** 10.36660/abc.20220707

**Published:** 2023-06-07

**Authors:** Tarso Augusto Duenhas Accorsi, Milena Ribeiro Paixão, José Leão de Souza, Marcus Vinicius Burato Gaz, Ricardo Galesso Cardoso, Karen Francine Köhler, Karine De Amicis Lima, Flavio Tarasoutchi, INTEGRATE Investigadores

**Affiliations:** 1 Unidade de Pronto Atendimento Hospital Israelita Albert Einstein São Paulo SP Brasil Unidade de Pronto Atendimento , Hospital Israelita Albert Einstein , São Paulo , SP – Brasil; 2 Instituto do Coração Faculdade de Medicina USP São Paulo SP Brasil Instituto do Coração (InCor), Faculdade de Medicina , USP , São Paulo , SP – Brasil

**Keywords:** Doença valvar cardíaca, valvas cardíacas, prótese de valvar cardíaca, medicina de emergência

## Abstract

A doença valvar cardíaca é um problema de saúde crescente no mundo. Os pacientes com valvopatia podem apresentar diversas emergências cardiovasculares. O manejo desses pacientes é um desafio no departamento de emergência, principalmente quando a condição cardíaca prévia é desconhecida. Atualmente, recomendações específicas para o manejo inicial são limitadas. A presente revisão integrativa propõe uma abordagem baseada em evidência, de três etapas, desde a suspeita de valvopatia à beira do leito até o tratamento inicial das emergências. A primeira etapa é a suspeita de uma condição valvar subjacente com base nos sinais e sintomas. A segunda etapa consiste na tentativa de confirmação diagnóstica e avaliação da gravidade da valvopatia com exames complementares. Finalmente, a terceira etapa aborda as opções diagnósticas e terapêuticas para insuficiência cardíaca, fibrilação atrial, trombose valvar, febre reumática aguda, e endocardite infecciosa. Além disso, apresentamos imagens de exames complementares e tabelas para apoio aos médicos.

## Introdução

A doença valvar cardíaca afeta 2,5% da população no mundo, com um aumento acentuado após a idade de 65 anos. ^1^ O curso natural da valvopatia geralmente culmina em insuficiência cardíaca (IC). ^1 - 4^ Embora a cirurgia cardíaca seja ainda o principal tratamento definitivo, avanços nas intervenções por cateter aumentaram as opções terapêuticas. ^5^ As diretrizes de valvopatias, apesar de recentemente atualizadas, são direcionadas aos pacientes crônicos, ^2 - 4^ com carência de recomendações específicas para apresentações agudas.

Os pacientes com valvopatia podem apresentar diversas emergências cardiovasculares, tais como IC aguda, arritmias, eventos trombóticos, endocardite infecciosa (EI) e febre reumática (FR) aguda. Quando a existência de doença valvar prévia é desconhecida, identificar a condição subjacente é ainda mais desafiador, principalmente por médicos não cardiologistas. ^6^ São necessários medicamentos e intervenções específicos, de acordo com cada valvopatia. ^2 - 4^ O objetivo desta revisão é realizar uma abordagem baseada em evidência, passo a passo, desde suspeita de valvopatia no Departamento de Emergência (DE) até o tratamento das emergências mais prevalentes.

### Abordagem em três etapas

Três etapas são recomendas desde a suspeita da valvopatia até o manejo das emergências cardiovasculares. A primeira etapa é reconhecer a possibilidade de valvopatia no DE e a etapa seguinte consiste em uma avaliação mais detalhada com exames complementares. Embora a ecocardiografia seja o principal exame diagnóstico de imagem, é provável que ela não esteja prontamente disponível. Por isso, seria essencial a identificação de sinais de valvopatia por métodos mais disponíveis no DE, como eletrocardiograma (ECG), radiografia do tórax e ultrassom *point-of-care* (POCUS). Sinais de alerta detectados nesses testes à beira do leito devem agilizar o diagnóstico ecocardiográfico e a avaliação de gravidade. Finalmente, a terceira etapa consiste em diagnosticar e implementar tratamentos iniciais específicos das principais emergências relacionadas à doença valvar cardíaca ( Figura Central ).

**Figura Central:**
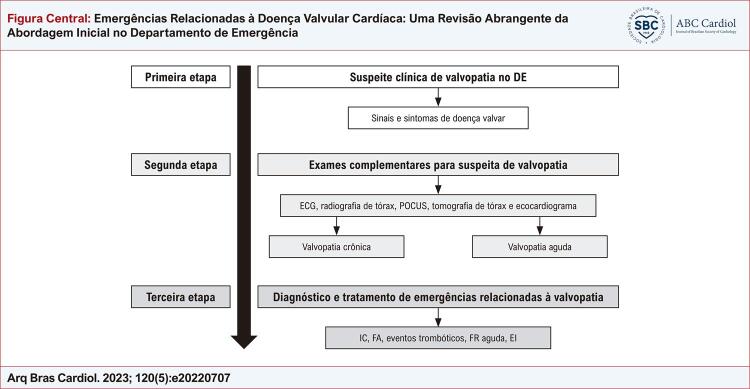
: Emergências Relacionadas à Doença Valvular Cardíaca: Uma Revisão Abrangente da Abordagem Inicial no Departamento de Emergência

Abordagem em etapas das emergências relacionadas à doença valvular cardíaca; DE: Departamento de Emergência; ECG: Eletrocardiograma; POCUS: ultrassom point-of-care; IC: Insuficiência Cardíaca; FA: Fibrilação Atrial; FR: Febre Reumática; EI: Endocardite Infecciosa.

### Primeira etapa: suspeita clínica de valvopatia no DE

A suspeita de doença valvar no DE origina-se da história clínica, mas a identificação de alterações no exame físico, principalmente a presença de sopro cardíaco, é o indicador mais importante. ^6 , 7^ Os sintomas cardiovasculares aparecem em estágios anatomicamente avançados da valvopatia como parte da história natural da doença valvar nativa ou de prótese valvar ( Figura Suplementar 1 ). Com menor frequência, os sintomas podem ainda ocorrer como um início agudo de doença valvar. Os principais sinais e sintomas relacionados à valvopatia estão resumidos na Tabela 1 . ^2 - 4 , 6 , 7^

**Tabela 1:** – Sinais e sintomas da doença valvar cardíaca

Sintomas	
Dispneia, ortopneia, dispneia paroxística noturna, edema Dor no peito Síncope Palpitação Eventos embólicos como acidente vascular cerebral	
Sinais	Valvulopatia
**Sopro:** – Holossistólico no ictus – Sistólico crescendo–decrescendo na borda esternal superior direita – Som diastólico no ictus, geralmente com um estalido de abertura – Diastólico decrescendo detectado geralmente no terceiro espaço intercostal à esquerda – Holossistólico, aumentando durante a inspiração na borda esternal inferior esquerda – Sopro médio diastólico curto, mais alto no ictus na ausência de um estalido de abertura, associado a um sopro holossistólico	Insuficiência mitral Estenose aórtica Estenose mitral Insuficiência aórtica Insuficiência tricúspide Valvulite mitral na FR aguda (Carey–Coombs)
**Pulso:** – Pulso em martelo d’água (pulso de Watson ou de Corrigan) – Pulso lento e tardio ( *parvus et tardus* )	Insuficiência aórtica Estenose aórtica
**Cabeça:** – Bochechas rosadas/arroxeadas (fácies mitral) – Movimento rítmico da cabeça seguindo os batimentos cardíacos (sinal de Musset) – Pulsação visível da úvula (sinal de Muller) – Pulsação visível das pupilas (sinal de Landolfi)	Estenose mitral Regurgitação aórtica
**Olhos:** – Hemorragia retiniana com centro pálido (manchas de Roth)	Endocardite infecciosa
**Pescoço:** – Pulsações sistólicas gigantes com onda V proeminentes (sinal de Lancisi) – Dança das carótidas (sinal de Corrigan)	Insuficiência tricúspide Insuficiência aórtica
**Dedos:** – Pulsação capilar à leve compressão da unha (sinal de Quincke) – Baqueteamento digital – Hemorragia Splinter	Insuficiência aórtica Cardiopatias congênitas, endocardite infecciosa Endocardite infecciosa
**Abdômen:** – Pulsação do baço (sinal de Gerhardt) e fígado (sinal de Rosenbach)	Insuficiência aórtica
**Pele:** – Nódulos subcutâneos – Eritema *marginatum* – Nódulos de Osler – Nódulos de Janeway – Petéquias	Febre reumática aguda Endocardite infecciosa

FR: febre reumática.

O ambiente de emergência limita a realização adequada de anamnese e exame físico, devido à ausência de privacidade, excesso de pessoas e barulho, e tempo limitado dedicado a cada paciente. ^8^ Além disso, sopro valvar, como regurgitação aórtica ou mitral aguda são geralmente pouco ou nada audíveis devido à pouca variabilidade de pressão entre as câmaras cardíacas. A transmissão dos sopros também pode ser afetada por estresse respiratório. ^9^ Assim, os médicos emergencistas devem sempre estar atentos à possibilidade de doença valvar nas emergências cardiovasculares.

### Segunda etapa: exames complementares na suspeita de doença valvar

As emergências relacionadas à valvopatia são principalmente constituídas de disfunção valvar grave, uma condição associada a múltiplas alterações anatômicas do coração. A avaliação à beira do leito do ECG, da radiografia do tórax, do POCUS e, em alguns casos, da tomografia do tórax, pode prever o diagnóstico de valvopatia. ^2 - 4^ Os exames padrões para as valvopatias agudas e crônicas mais prevalentes estão descritos abaixo. Os principais achados ecocardiográficos foram resumidos de modo que os médicos emergencistas possam consulta-los usando o POCUS e dar atenção extra a esses dados à avaliação do laudo ecocardiográfico.

### Doença valvar crônica importante

#### Estenose aórtica crônica importante

**Figura 1:**
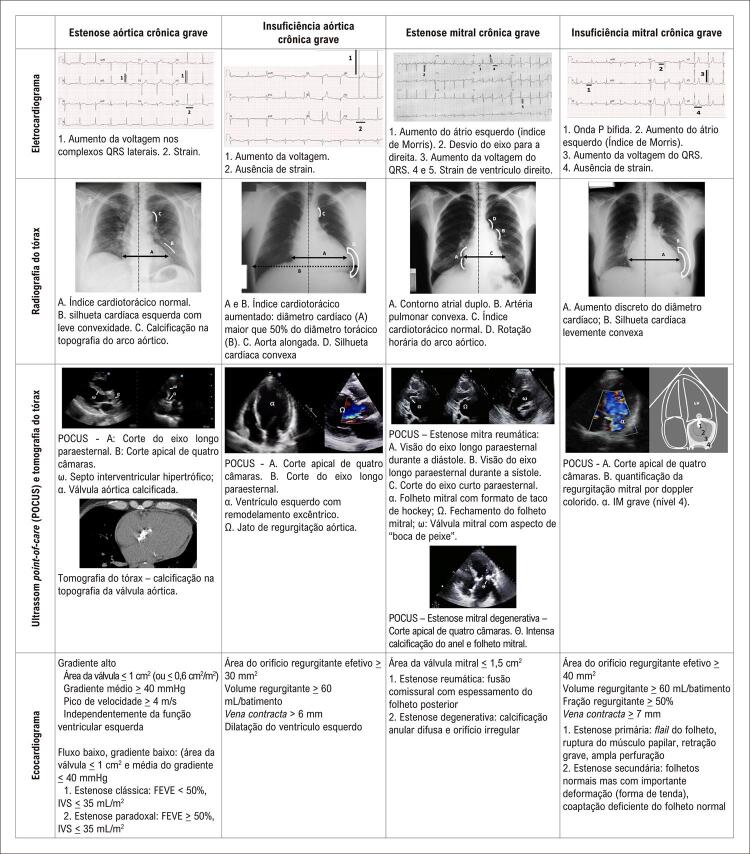
– Achados de imagens de doença valvular cardíaca. FEVE: fração de ejeção do ventrículo esquerdo; IVS: índice de volume sistólico; IM: insuficiência mitral.

A estenose aórtica (EA) importante induz hipertrofia concêntrica do ventrículo esquerdo (VE) que pode ser detectada no ECG, na radiografia do tórax, e no POCUS. ^2 , 10^ O POCUS também pode revelar valva aórtica calcificada com mobilidade reduzida. ^10^ A detecção tomográfica e a quantificação de calcificação aórtica é um marcador valioso de EA importante. ^11^ O fator mais importante no ecocardiograma é a redução da área valvar aórtica ( Figura 1 ). ^2 , 12^

#### Insuficiência aórtica crônica importante

O remodelamento excêntrico do VE é a principal característica da insuficiência aórtica (IA), facilmente detectada por exames realizados à beira do leito. ^13^ Os critérios ecocardiográficos baseiam-se em medidas quantitativas do jato regurgitante ( Figura 1 ). ^2 , 12^

#### Estenose mitral crônica importante

A estenose mitral (EM) é uma causa de IC sem sobrecarga do VE. Imagens da EM importante podem mostrar sobrecarga atrial esquerda, hipertensão pulmonar, e remodelamento secundário das câmaras direitas. ^14^ Os critérios ecocardiográficos são baseados na redução da área valvar e padrão de calcificação ( Figura 1 ). ^2 , 12^

#### Insuficiência mitral crônica importante

A insuficiência mitral (IM) é uma das valvopatias mais prevalentes. A IM grave geralmente apresenta sobrecarga atrial esquerda e um remodelamento excêntrico moderado do VE. ^15^ Os critérios ecocardiográficos baseiam-se nas medidas quantitativas do jato regurgitante ( Figura 1 ). ^2 , 12^ Além de avaliar a gravidade, para um tratamento em longo prazo, é importante diferenciar a IM primária da IM secundária.

#### Doença valvar aguda importante

Quase todas as doenças valvares agudas são IM ou IA. ^9 , 13 , 15^ As principais causas são EI, FR, lesão relacionada a procedimentos (valvoplastia percutânea com balão ou cateterismo cardíaco), ruptura espontânea da prótese, e contusão. ^16 , 17^ A IA também pode ocorrer em dissecção aórtica tipo A e IM na síndrome coronariana aguda (tracionamento ou “tethering” dos folhetos), ruptura de corda tendínea, e cardiomiopatia aguda (como síndrome de takotsubo, miocardiopatia periparto e miocardiopatia viral). ^17^

Não há achados típicos de valvopatia aguda no ECG. Geralmente observa-se congestão pulmonar na radiografia de tórax e no POCUS. Embora um edema pulmonar assimétrico no logo superior do pulmão direito possa ser causado por IM aguda, mesmo esse padrão de congestão não é suficiente para definir a valvopatia. ^16^ Assim, o ecocardiograma, é a estratégia mais precisa para diagnosticar uma valvopatia aguda. ^16 - 18^

## Avaliação complementar

O peptídeo natriurético cerebral (BNP) é um biomarcador prognóstico preciso em pacientes com doenças cardíacas como a IC. Contudo, os níveis plasmáticos de BNP estão geralmente dentro da faixa de normalidade em pacientes com IC de causa valvar apesar do remodelamento cardíaco. ^19^ Níveis mais altos de BNP em pacientes com estenose aórtica, regurgitação aórtica e IM estão associados com aumento no átrio esquerdo, na pressão pulmonar, redução na capacidade do exercício, e pior prognóstico. ^4 , 19^ Consequentemente, embora as concentrações de BNP não sejam confiáveis na identificação de valvopatia importante, seus níveis plasmáticos elevados podem sugerir piores desempenho físico e desfechos.

Pacientes com valvopatia apresentam fração de ejeção do VE (FEVE) reduzida em condições crônicas avançadas, principalmente na insuficiência aórtica e mitral. O POCUS é usado para avaliar a função sistólica do VE. A impressão subjetiva de uma contração deficiente do VE ( Figura Suplementar 2 ) tem uma correlação significativa com a FEVE na ecocardiografia. No DE de um centro brasileiro de cardiologia, 27,5% dos pacientes com IC secundária à valvopatia apresentaram IM, 23% apresentaram EA 13% IA e 11% EM. ^20^

A ausculta pulmonar pode ser normal em pacientes com valvopatia, mesmo com congestão pulmonar importante. Neste cenário, o POCUS pulmonar tem uma alta razão de verossimilhança positiva no diagnóstico de IC quando pelo menos três linhas B são identificadas em um plano longitudinal entre duas costelas em duas ou mais regiões bilateralmente ( Figura Suplementar 3 ). ^21^

## Terceira etapa: diagnóstico e tratamento de emergências relacionadas à valvopatia

O tratamento de pacientes com emergências relacionadas às valvopatias têm diversas particularidades. A principal abordagem para o diagnóstico e tratamento de IC, fibrilação atrial (FA), trombose valvar, FR, e EI será discutida a seguir.

Para essa etapa, o médico emergencista precisa assegurar-se de que tanto o paciente como outros médicos especialistas participem do processo de tomada de decisão. Uma discussão multidisciplinar deveria ser encorajada em todos os centros, principalmente para decisões terapêuticas críticas. Cardiologistas clínicos e intervencionistas, ecocardiografistas, cirurgiões cardíacos, infectologistas, anestesiologistas, e radiologistas geralmente fazem parte da “equipe do coração” ( *Heart Team* ). Outros profissionais podem ser necessários em casos mais complexos. ^2 - 4^

## Insuficiência cardíaca

A IC progressiva em pacientes com valvopatia crônica é a principal causa de atendimento de emergência. ^22^ Os principais achados na IC por valvopatia são os mesmos que na IC por outras causas: dispneia, ortopneia, taquicardia, impulso apical anormal, pressão arterial sistólica baixa, distensão da veia jugular, e edema. Em caso de início repentino ou rápida progressão do edema pulmonar e instabilidade hemodinâmica, a valvopatia aguda é mais comum. ^16 , 17^ Mesmo em pacientes sem hipotensão, o choque cardiogênico deve ser considerado naqueles que apresentem fadiga, fraqueza, tontura, diminuição da consciência, síncope, aumento na frequência cardíaca ou na taxa respiratória, livedo, e história de redução na diurese. ^23 - 25^

A doença valvar cardíaca tem diferentes mecanismos hemodinâmicos. Assim, o manejo é individualizado com base na fisiopatologia de cada doença. ^2^ O trabalho do VE pode ser representado por uma curva de pressão e volume (PV) ( Figura Suplementar 4 ). ^26^ O VE normal apresenta ciclos de baixa pressão e complacência adequada. ^27^ Uma vez que o débito cardíaco depende da pré-carga, da pós-carga, e do inotropismo, mudanças nesses parâmetros afetam a curva PV. Tanto a valvopatia aguda como a valvopatia crônica modificam a curva PV. Por exemplo, 1) devido ao desvio para a direita da curva de volume no VE na EM, medidas para aumentar o inotropismo tem pouco benefício na presença de baixo débito; 2) na EA, existe elevada pressão do VE; portanto, uma redução substancial no volume do VE com terapia diurética pode induzir um baixo débito; 3) a vasodilatação em lesões regurgitantes é essencial para reduzir a pressão do enchimento ventricular e aliviar a congestão. ^28^ O objetivo do tratamento medicamentoso é reajustar esses parâmetros até o tratamento invasivo definitivo. Assim, medicamentos e tratamentos específicos são necessários para cada valvulopatia, geralmente diferente dos aplicados em outras causas de IC.

Na EA importante, devido à presença de um débito cardíaco fixo, a principal terapia é o uso de diuréticos. Devem-se evitar betabloqueadores e vasodilatação devido à possibilidade de diminuição do débito cardíaco. O manejo do choque cardiogênico inclui algumas precauções: evitar a taquicardia causada por drogas vasoativas, evitar fluidoterapia já que a maioria dos pacientes são hipervolêmicos, e considerar o uso de balão intra-aórtico, oxigenação por membrana extracorpórea, e valvuloplastia aórtica percutânea por balão como estratégias temporárias para o controle hemodinâmico como ponte para uma intervenção definita. ^2 , 24 , 25 , 29 , 30^

A IM e IA beneficiam-se de vasodilatação e diuréticos, geralmente com boa resposta clínica. ^2 , 31 - 33^ Betabloqueadores e bloqueadores de canais de cálcio não fazem parte da terapia padrão e são usados na tentativa de se controlar a frequência cardíaca em pacientes com FA de alta resposta ventricular. ^2^ O balão intra-aórtico é contraindicado na regurgitação aórtica, uma vez que ele acentua a disfunção valvar e diminui o débito cardíaco. Há evidência de que o dispositivo possa ser benéfico na EM, por exemplo, como suporte temporário para pacientes com ruptura papilar após infarto do miocárdio até a cirurgia. ^23 , 34^

A EM é a única valvopatia em que betabloqueadores e bloqueadores de canal de cálcio fazem parte da terapia principal. ^2 , 35 - 37^ A ivabradina pode ser usada em ritmo sinusal como uma alternativa para pacientes intolerantes a betabloqueadores ou combinada a eles se a frequência cardíaca se mantiver acima de 60 bpm. ^36^ A digoxina é a opção para pacientes com FA, e os diuréticos podem ser úteis no manejo da congestão. ^2 , 35 - 38^ No edema agudo dos pulmões, a terapia de primeira linha inclui controle da frequência cardíaca e diuréticos. Nesse caso, se o paciente apresenta disfunção ventricular direita confirmada ou presumida (sinais e sintomas de IC direita), deve-se preferir o uso de digitálico em relação aos betabloqueadores para a manutenção da contratilidade do miocárdio, e ventilação invasiva ou não invasiva deve ser evitada, uma vez que uma pressão torácica aumentada resulta em diminuição da pré-carga ventricular direita. ^36^ Durante a gravidez, o aumento fisiológico no volume sanguíneo e na frequência cardíaca impõe um maior risco de descompensação, mesmo em mulheres previamente assintomáticas. Para essa população, as principais opções farmacológicas são o propranolol ou o metoprolol, e a digoxina. A valvuloplastia mitral percutânea por balão pode ser realizada em mulheres grávidas e em pacientes refratários à terapia medicamentosa quando a anatomia for favorável. ^24 , 39^

Indicações detalhadas para a hidratação, vasopressores, balão intra-aórtico, outras estratégias intervencionistas de suporte, ventilação não invasiva, manejo avançado das vias aéreas, diuréticos, e otimização da curva VP do VE estão detalhadas na Tabela 2 ^2 - 4 , 23 - 25 , 29 , 31 - 51^ As doses de medicamentos mais frequentemente empregadas são descritas na Tabela Suplementar 1. ^2 - 4 , 23 - 25 , 29 , 31 - 33 , 35 - 37 , 47 - 50^

**Tabela 2:** – Manejo hemodinâmico no departamento de emergência

Manejo da perfusão orgânica em pacientes hipotensos				
	Estenose aórtica	Insuficiência aórtica	Estenose mitral	Insuficiência mitral
Fluidoterapia: Cristaloides Isotônicos Evitar soluções hipertônicas e hipotônicas Não há fortes evidências para expansor de volume coloidal.	Atenção: a maioria dos pacientes apresenta congestão. A fluidoterapia não é apropriada e pode induzir desconforto respiratório. Os fluidos devem ser administrados em caso de sinais ultrassonográficos inequívocos de hipovolemia.			
	FEVE normal + “ *kissing walls”* + VCI < 10mm + ausência de linhas B Altamente dependente da pré-carga	FEVE normal + VCI < 10mm + ausência de linhas B	FC < 100 bpm (geralmente em uso de medicação) + diâmetro do VD < diâmetro do VE + ausência de formato de “D” + VCI < 10 mm + ausência de linhas B	FEVE + diâmetro de VD < diâmetro do VE + ausência de formato de “D” + VCI < 10 mm + ausência de linhas B
Vasopressores	Dobutamina em dose baixa– prevenir taquicardia	Dobutamina	Evitar dobutamina (prevenir taquicardia)	Dobutamina
	Norepinefrina em dose baixa – prevenir taquicardia	Norepinefrina	Norepinefrina em dose baixa – prevenir taquicardia	Norepinefrina
Outras drogas	Aminas vasoativas de ação curta podem ser usadas (fenilefrina)	Sem evidência forte	Em caso de taquicardia (mesmo se em ritmo sinusal), betabloqueadores podem ser usados; aminas vasoativas de ação curta podem ser usadas short- (fenilefrina). Milrinona se hipertensão pulmonar.	Milrinona se hipertensão pulmonar
Balão intra-aórtico	Possível benefício	Contraindicado	Sem evidência	Possível benefício
Intervencionista / Outras estratégias	Considerar valvuloplastia com balão percutâneo como ponte para a intervenção definitiva: oxigenação por membrana extracorpórea como ponte a intervenção definitiva	Não há forte evidência	Considerar valvuloplastia mitral percutânea com balão se houver critérios favoráveis (anatômicos e ausência de contraindicação)	Dispositivo percutâneo de assistência ventricular como ponte para intervenção definitiva. Não há evidência atual para uso do MitraClip
**Manejo da hipoxemia**				
	**Estenose aórtica**	**Insuficiência aórtica**	**Estenose mitral**	**Insuficiência mitral**
Ventilação não invasiva – evitar sedação profunda e opioides	Possível, mesmo na hipotensão leve	Possível, mesmo na hipotensão leve	Possível, mesmo na hipotensão leve; evitar na hipertensão pulmonar e/ou disfunção ventricular direita	Possível, mesmo na hipotensão leve; evitar na hipertensão pulmonar e/ou disfunção ventricular direita
Manejo avançado das vias aéreas; não há fortes evidências para pré-medicação com lidocaína. Estratégias de sedação (escolher um): Propofol Etomidato Ketamina Midazolam mais (escolher um): Succinilcolina Rocurônio Ventilação mecânica inicial: volume corrente 6 mL/Kg, pressão de platô < 30 mmHg, PEEP titulada e pressão motriz ( *driving pressure* )< 20 mmHg	A hipotensão geralmente ocorre após a intubação; atenção à escolha dos medicamentos durante a sedação e manter os vasopressores disponíveis	Intubação é geralmente bem tolerada	Evitar cetamina; hipotensão importante após intubação quando há hipertensão pulmonar	Intubação é geralmente bem tolerada
Diuréticos (furosemida; sem evidência de outras classes no departamento de emergência)	Administrar somente em caso de congestão pulmonar; evitar em caso de oxigenação compensada	Geralmente necessário	Geralmente necessário	Geralmente necessário
**Otimizar curva pressão x volume até o tratamento definitivo**				
	**Estenose aórtica**	**Insuficiência aórtica**	**Estenose mitral**	**Insuficiência mitral**
Ritmo A amiodarona pode ser usada em todos os cenários de acordo com a avaliação clínica em pacientes estáveis com arritmia supraventricular aguda.	Manter ritmo sinusal se possível	Considerar FA estável crônica como taquicardia sinusal. Diltiazem e esmolol podem ser usados com cuidado.	Manter ritmo sinusal se possível	Considerar FA estável crônica como taquicardia sinusal. Diltiazem e esmolol podem ser usados com cuidado.
Frequência cardíaca	Evitar taquicardia excessiva em ritmo não sinusal com amiodarona, diltiazem, verapamil, esmolol, tartarato de metoprolol, lanatosídeo C	Evitar uso rotineiro de betabloqueadores. Diltiazem e esmolol podem ser usados com cuidado.	Evitar taquicardia excessiva em todos os ritmos com amiodarona, diltiazem, verapamil, esmolol, tartarato de metoprolol, lanatosídeo C	80–100 bpm Evitar redução intensa. Diltiazem e esmolol podem ser usados com cuidado.
Pré-carga: POCUS deve monitorar VCI e outros parâmetros dinâmicos	Evitar diuréticos e nitratos (nitroglicerina, isossorbida)	Em pacientes estáveis com congestão pulmonar, é razoável utilizar vasodilatadores independentemente da classe – nicardipina, hidralazina, captopril e enalapril	Evitar uso rotineiro de vasodilatadores	Em pacientes estáveis com congestão pulmonar, é razoável utilizar vasodilatadores independentemente da classe – nicardipina, hidralazina, captopril e enalapril
Pós-carga	Nitroprussiato se PAM > 60 mmHg, principalmente se a FEVE for baixa; evitar uma rápida redução do volume sistólico (piora da perfusão coronária)	Nitroprussiato. Nitroglicerina deve ser uma alternativa. Em pacientes estáveis com congestão pulmonar, é razoável utilizar vasodilatadores independentemente da classe – nicardipina, hidralazina, captopril e enalapril	Evitar pós-carga baixa (reduz perfusão coronariana)	Prevenir aumento; Nitroprussiato. Nitroglicerina deve ser uma alternativa. Em pacientes estáveis com congestão pulmonar, é razoável utilizar vasodilatadores independentemente da classe – nicardipina, hidralazina, captopril e enalapril
Contratilidade	Levosimendan	Não há forte evidência	Não há forte evidência	Evitar depressão miocárdica

FEVE: fração de ejeção do ventrículo esquerdo; VCI: veia cava inferior; FC: frequência cardíaca; PAM: pressão arterial Média; PEEP: pressão expiratória final positiva; FA: fibrilação atrial; VD: ventrículo direito; VE: ventrículo esquerdo.

Cardiologistas clínicos e intervencionistas devem fazer parte da equipe para se discutir a melhor prática nesses casos. Na ausência de controle de sintomas com tratamento medicamentosos em centros não especializados, os pacientes devem ser imediatamente encaminhados para um centro de cardiologia para tratamentos especializados, como balão intra-aórtico, valvulopastia com balão e cirurgia cardíaca.

### Fibrilação atrial

A valvopatia pode se apresentar com arritmias, principalmente FA. A possibilidade de valvopatia deve ser considerada em cada cenário de FA no DE, especialmente em pacientes com instabilidade hemodinâmia. ^52^ Mais de 30% dos pacientes com FA apresentam valvopatia. ^53^

A cardioversão da FA no DE é realizada exclusivamente se a instabilidade do paciente for causada por arritmia. Na maioria dos casos, a abordagem mais segura é o controle da frequência cardíaca e o início da anticoagulação se indicada. A fim de se prevenir previnir a embolização induzida por cardioversão, o procedimento é recomendado após exclusão de trombo atrial por ecocardiograma transesofágico ou após três semanas de anticoagulação adequada. ^54^

Em longo prazo, existe um benefício em se manter o ritmo sinusal sempre que possível. O tamanho atrial não é utilizado para contraindicar a cardioversão; contudo, quanto mais significativo o remodelamento, menor a chance de reversão da FA e manutenção do ritmo sinusal. Outros fatores de risco de recorrência na FA incluem idade, disfunção renal, e outros fatores de risco cardiovasculares. ^4 , 54^

O acidente vascular cerebral é o evento mais temido em pacientes com FA. ^54 , 55^ A valvopatia é responsável por cerca de um terço dos casos de acidente vascular cerebral isquêmico entre 15 e 45 anos de idade. ^56^ Por isso, recomenda-se a avaliação dos riscos potenciais e os benefícios da anticoagulação para pacientes com valvopatia e FA. Os antagonistas da vitamina K são a escolha para prótese mecânica (o estudo RE-ALIGN foi interrompido prematuramente por eventos no grupo com que recebeu dabigatrana) ^57^ e EM (estudo INVICTUS). ^58^ Os anticoagulantes orais não antagonistas de vitamina K são recomendados em outras valvopatias, inclusive em pacientes com próteses biológicas (estudo RIVER). ^2 - 4 , 59^ O estudo PROACT Xa comparando o uso de apixabana com varfarina para prótese mecânica aórtica On-X foi interrompido por maior incidência de eventos no grupo apixabana. ^60^ Após FA, o controle inicial da anticoagulação por ser conduzida em seguimento ambulatorial, mesmo para pacientes em uso de antagonistas da vitamina K. Para pacientes com risco trombótico aumentado e baixo risco de sangramento, o uso de enoxaparina pode ser considerado em pacientes em uso de antagonistas da vitamina K até se atingir o objetivo terapêutico. Essa terapia transitória não se aplica para pacientes em uso de anticoagulantes diretos. ^61^

### Trombose valvar

A trombose de prótese é caracterizada pela formação de um trombo nas estruturas da prótese, com subsequente disfunção da valva, com ou sem tromboembolismo. ^62^ Ocorre mais comumente em próteses mecânicas, variando entre 0,1% e 5,7%, principalmente no período perioperatório precoce, na posição mitral e anticoagulação subterapêutica. ^63^

A trombose de prótese pode manifestar-se de diferentes maneiras, dependendo da gravidade da disfunção da válvula, e do tamanho e mobilidade do trombo. Os pacientes podem ser assintomáticos com trombo detectado incidentalmente e, em outros casos, o trombo pode causar embolia, hipotensão, síncope, dispneia, congestão pulmonar e morte súbita. ^64 , 65^ O diagnóstico é classicamente confirmado por ecocardiograma transesofágico, mas a avaliação por tomografia computadorizada multidetectores e radioscopia pode ser útil.

A trombose de prótese não obstrutiva em pacientes estáveis é tratada por meio da otimização da anticoagulação oral. A fibrinólise ou a cirurgia é recomendada para pacientes com trombo remanescente após anticoagulação ótima ou com trombo ≥ 10mm e/ou >0,8cm ^2^ associada com êmbolos. Pacientes hemodinamicamente instáveis devem ser prontamente submetidos à substituição de valva. Para pacientes com risco elevado ou proibitivo, a fibrinólise é a principal opção. A fibrinólise é realizada com alteplase 90 mg em 90 minutos ou com estreptoquinase 1,500.000 UI em 60 minutos. Complicações potenciais desse tratamento são sangramento, embolia, e recorrência de trombose. ^2 - 4 , 66^ Esses são pacientes de alto risco e, por isso, a tomada de decisão deve envolver o cardiologista clínico e o cirurgião cardíaco.

### Febre reumática

A FR é uma resposta autoimune à infecção da faringe por Streptococcus do grupo A, que ocorre de duas a quatro semanas após a exposição. Indivíduos geneticamente susceptíveis correspondem a 0,1-5% da população. ^67 - 69^ O primeiro episódio geralmente manifesta-se em crianças em idade escolar em regiões de baixa renda. ^67 - 70^ Fatores ambientais relacionados a níveis elevados de infecção por Streptococcus são alta concentração de pessoas no domicílio, saneamento precário, e menor uso de antibióticos para tratar faringite. ^71^ A apresentação clínica está resumida na Tabela 3 . ^67 , 68^ Não existe nenhum exame laboratorial diagnóstico para FR; o diagnóstico deve preencher os critérios de Jones ( Tabela 4 ). ^67 , 68 , 72^

**Tabela 3:** – Manifestações clínicas de febre reumática aguda

Manifestação	Prevalência (%)	Sinais e sintomas
Cardite	50–70	Valvulite é a principal manifestação, embora miocardite e pericardite possam também acontecer. Em caso de valvulite grave, pode ocorrer insuficiência cardíaca. A cardite subclínica refere-se ao diagnóstico ecocardiográfico na ausência de achados auscultatórios.
Artrite	35–66	Poliartrite migratória, com duração de um a sete dias em cada articulação; articulações maiores são mais afetadas: joelhos, tornozelos, cotovelos e punhos. A dor é mais intensa que os achados clínicos. Ausência de deformidade em longo prazo. O início precoce de AINES pode induzir monoartrites devido a seu rápido desenvolvimento.
Coreia	10–30	Movimentos involuntários, abruptos, não ritmados do tronco, extremidades, cabeça, face e língua, relacionados à labilidade emocional e fraqueza muscular. Desaparece durante o sono. Geralmente inicia-se três meses após infecção por Streptococcus do grupo A e dura entre dois e três meses
Nódulos subcutâneos	0–10	Nódulos firmes, indolores, medindo até 2 cm, geralmente três ou quatro, localizados sobre um osso ou superfície extensora dos tendões (geralmente o olecrano), com pelo normal ao redor. Surge uma a duas semanas após outras manifestações e dura menos de um mês. Relacionados à cardite: quase nunca é a única manifestação de FR.
Eritema *marginatum*	< 6	*Rash* rosa, arredondado, com centro pálido, ou com bordas serpiginosas no tronco ou extremidades proximais (face preservada). As lesões clareiam sob pressão e podem aparecer, desaparecer, e reaparecer em horas. Relacionada à cardite: quase nunca é a única manifestação de FR.

FR: febre reumática; AINES: anti-inflamatórios não esteroides.

**Tabela 4:** – Critérios diagnósticos atuais para febre reumática

	Grupos de alto risco (residentes de áreas endêmicas; histórico pessoal; idade <40 anos; residência atual, frequente ou recente em áreas de alto risco de doença reumática)	Grupos de baixo risco (sem condições de alto risco)
Manifestações maiores	cardite (incluindo evidência subclínica de valvulite no ecocardiograma) poliartrite ou monoartrite asséptica ou poliartralgia Coreia de Sydenham eritema *marginatum* e) nódulos subcutâneos	cardite (incluindo evidência subclínica de valvulite no ecocardiograma) poliartrite Coreia de Sydenham eritema *marginatum* nódulos subcutâneos
Manifestações menores	Febre > 38 ^o^ C Monoartralgia taxa de sedimentação de eritrócitos > 30 mm/h ou proteína C reativa > 30 mg/L d) Intervalo PR prolongado no ECG	Febre > 38,5 ^O^ C poliartrite ou monoartrite asséptica taxa de sedimentação de eritrócitos > 60 mm/h ou proteína C reativa > 30 mg/L Intervalo PR prolongado no ECG
Episódio inicial de FR aguda	2 manifestações maiores + evidência de infecção prévia por SGA; ou 1 manifestação maior + 2 manifestações menores + evidência de infecção prévia por SGA	
Episódio recorrente de FR aguda em pacientes com histórico de FR ou DCR	2 manifestações maiores + evidência de infecção prévia por SGA; ou 1 manifestação maior + 2 manifestações menores + evidência de infecção prévia por SGA; ou 3 manifestações menores + evidência de infecção prévia por SGA	
FR aguda provável ou possível (inicial ou recorrente)	1 manifestação maior ou 1 manifestação menor; ou sem evidência de infecção prévia por SGA. FR aguda provável (e não suspeita) FR aguda possível (e não incerta)	

FR: febre reumática; SGA: staphylococcus do grupo A; DCR: doença cardíaca reumática; ECG: eletrocardiograma.

O tratamento de FR aguda tem três pilares além daqueles mencionados para valvopatia: a erradicação da infecção por Streptococcus, administração de medicamentos de acordo com as manifestações e profilaxia ( Tabela 5 ). ^67 , 68 , 73^

**Tabela 5:** – Tratamento da febre reumática

	Tratamento
Todos os casos	Erradicação da infecção por SGA: – Penicilina benzatina 1.200.000 unidades (criança < 20kg: 600.000 unidades; ≥ 20kg: 1.200.000 unidades) dose única intramuscular – Hipersensibilidade à penicilina: cefalexina 1 g (criança: 25 mg/kg até 1 g) via oral, a cada 12 horas por 10 dias ou azitromicina 500 mg (criança: 12 mg/kg até 500 mg) via oral por 5 dias
Cardite	Repouso, com mobilização conforme os sintomas Prednisona/prednisolona 1–2 mg/kg até uma dose máxima de 80 mg via oral, uma vez ao dia ou em doses divididas, por 4 – 8 semanas para cardite leve e 12 semanas em caso de cardite moderada/grave. Após 2-3 semanas, se os sintomas melhorarem, reduzir a dose em 20 a 25% semanalmente. Para casos refratários, 30 mg/kg/dia semanalmente Dose pediátrica: furosemida 1–2 mg/kg via oral como dose única, em seguida 0,5–1 mg/kg (até 6 mg/kg) via oral a cada 6-24 horas; espironolactona 1–3 mg/kg (inicialmente) até 100 mg via oral, diariamente em 1-3 doses (dose aproximadas de múltiplos de 6,25 mg – um quarto de um tablete de 25 mg); enalapril 0,1 mg/kg via oral, diariamente em 1 ou 2 doses, com aumento gradual em duas semanas para dose máxima de 1 mg/kg via oral, diariamente em 1 ou 2 doses, outros inibidores de ECA (captopril, lisinopril, ramipril, perindopril) podem ser usados. Dose adulta: Furosemida 20–40 mg oral ou intravenosa como dose única seguido de 20–40 mg oral ou intravenosa a cada 8–12 horas; espironolactona pode ser usada para pacientes com resposta limitada ou ausente a diuréticos de alça, espironolactona 12,5–25 mg oral diariamente; nitrato pode ser adicionado àqueles pacientes com resposta limitada ou ausente a diuréticos de alça, cuja pressão arterial sistólica é maior que 90 mmHg; inibidor de ECA é recomendado para pacientes com disfunção sistólica do ventrículo esquerdo moderada ou grave, a menos se contraindicado. Cirurgia valvar para cardite fatal (raro)
Artrite	Duração de cerca de um mês sem tratamento Rápida melhora com AINES, geralmente de 1 a 2 semanas (pode ser até 12 semanas) – Naproxeno de liberação imediata 250–500 mg (criança 10–20 mg/kg/dia) via oral duas vezes ao dia,até o máximo de 1250 mg/dia – Ibuprofeno 200–400 mg (criança 5-10 mg/kg) vira oral três vezes ao dia, até o máximo de 2400 mg/dia – Aspirina: adultos e crianças 50–60 mg/kg/dia vira oral em 4-5 doses; a dose pode ser aumentada até o máximo de 80–100 mg/kg/dia em 4-5 doses Inibidor de bomba de próton deve ser considerado Corticosteroides são usados em caso de intolerância ou alergia a AINES ou para tratamento de outra manifestação (cardite)
Coreia	Sem tratamento farmacológico para casos leves; para casos moderados/graves: – Haloperidol 0,5 mg por dose via oral duas vezes/dia (pode ser aumentada 0.5 mg/dia até 5mg/dia) – Carbamazepina 3,5–10 mg/kg por dose via oral duas vezes/dia – Valproato de sódio 7,5–10 mg/kg por dose via oral duas vezes/dia Considerar a adição de corticosteroides: prednisona/prednisolona 1–2 mg/kg até a dose máxima de 80mg via oral uma vez por dia ou em doses divididas
Profilaxia	Penicilina g benzatina intramuscular – Adultos > 20 kg: 1.2 milhões unidades a cada 21 a 28 dias – Crianças ≤ 20 kg: 600000 unidades a cada 21 a 28 dias Em caso de alergia à penicilina: – Sulfadiazina: < 30 kg 500 mg via oral uma vez ao dia; ≤ 30 kg: 1 g via oral, uma vez ao dia – Eritromicina: 250 mg via oral duas vezes ao dia Febre reumática sem cardite: por cinco anos ou até a idade de 21 anos (o que for mais longo) Febre reumática com cardite sem doença cardíaca residual (sem evidência clínica ou ecocardiográfica de doença valvar): por 10 anos ou até a idade de 21 anos (o que for mais longo) Febre reumática com cardite com doença cardíaca residual (doença valvar persistente): por 10 anos ou até a idade de 40 anos (o que for mais longo); em alguns casos, profilaxia por toda a vida (i.e., profissionais de saúde, professores de ensino infantil)

SGA: staphylococcus do grupo A; ECA: enzima conversora de angiotensina; AINES: anti-inflamatórios não esteroides.

### Endocardite infecciosa

A EI é uma infecção da valva cardíaca nativa ou prostética, endocárdio ou do dispositivo cardíaco implantado. Apesar dos avanços das estratégias diagnósticas e terapêuticas, a mortalidade em um ano continua em 30%. ^74^

O diagnóstico deve ser considerado em pacientes sem uma presença clara de infecção com foco nas avaliações clínicas, bem como na presença de sopro cardíaco ou condição cardíaca predisponente combinada a uma das seguintes condições: febre, suspeita de embolia sistêmica, e IC aguda/subaguda. Outros sinais e sintomas como artralgia, mialgia, anorexia, perda de peso, sudorese noturna, calafrios, cefaleia, dor abdominal, dor nas costas, dispneia, e hematúria também podem estar presentes. As principais condições cardíacas predisponentes são valvopatia pré-existente, história de EI e uso de drogas intravenosas. Algumas manifestações clínicas podem aumentar a suspeita de EI, como manchas de Roth – hemorragia retiniana com centro esbranquiçado, nódulos de Osler – nódulos dolorosos na ponta dos dedos das mãos e dos pés, lesões de Janeway – lesões eritematosas ou hemorrágicas maculares ou nodulares não dolorosas nas palmas das mãos ou plantas dos pés, hemorragia Splinter – hemorragia não dolorosa no terço distal da unha e petéquia na pele e membranas mucosas. ^75 - 78^

A EI tem apresentação clínica variada. Os critérios anatomopatológicos são obtidos em somente 20-40% dos casos. Os critérios de Duke são um conjunto de critérios diagnósticos para EI, mas suas principais limitações são o fato de não serem imediatos, e a acurácia na admissão variar entre 52% e 70% (Tabela Suplementar 2). ^75 - 78^

Os dois pilares do tratamento da EI são antibióticos ( Tabela 6 ) e cirurgia (Tabela Suplementar 3). ^75 - 80^ No DE, os antibióticos são fornecidos empiricamente com base no tipo de valvopatia e na EI. ^75 , 76^ Os objetivos primários da cirurgia incluem exploração, desbridamento e reconstrução ou substituição da valva/prótese. ^79^ Em geral, a realização da cirurgia somente após o término da terapia com antibióticos está associada com uma frequência mais alta do desfecho combinado de morte, eventos embólicos e recorrência de EI nos primeiros anos após a primeira infecção. Portanto, o tratamento cirúrgico deve ser considerado desde o início do tratamento e avaliado constantemente. ^75 - 80^

**Tabela 6:** – Antibioticoterapia empírica para endocardite infecciosa no departamento de emergência

Tipo de endocardite infecciosa	Antibióticos (regime e dose)	
Endocardite de válvula nativa ou de prótese valvar (> um ano de cirurgia)	Ampicilina 2 g IV a cada 4 horas + Oxacilina 2 g IV a cada 4 horas + Gentamicina* 1 a 3 mg/Kg de peso atual IV a cada 24 horas ou divido em três doses Alternativa em casos de baixa probabilidade de enterococci: ceftriaxona 1g a cada 12 horas em vez de ampicilina + oxacilina 2g IV a cada 4 horas	4 - 6 semanas** 4 - 6 semanas ** 2 semanas 4 - 6 semanas
Prótese valvar (< 1 ano da cirurgia)	Vancomicina 15 mg/Kg IV a cada 12 horas ou Daptomicina 8 to 10 mg/Kg IV a cada 24 horas Meropenem 2 g IV a cada 8 horas ou Cefepime 2 g IV a cada 8 horas + Gentamicina* 1 - 3 mg/Kg de peso atual IV a cada 24 h ou dividida em 3 doses	6 semanas 6 semanas 2 semanas

IV: intravenosa; * exceto em doença renal crônica ou nefrotoxicidade; **4 semanas para válvula nativa e 6 semanas para prótese valvar.

Diferentes profissionais devem estar envolvidos no tratamento do paciente. Além do emergencista, enfermeiros, infectologistas, cardiologistas e cirurgiões cardíacos, a equipe de endocardite pode também necessitar de um neurocirurgião ou cirurgião vascular (aneurisma micótico, embolização séptica), cirurgião geral ou gastrointestinal (abscesso de órgãos intra-abdnominais), ortopedista (discite), nefrologista (toxicidade do antibiótico), entre outros.

## Conclusão

Apesar da alta complexidade e heterogeneidade das emergências relacionadas às valvopatias, a abordagem em três etapas pode ajudar no raciocínio clínico. As etapas têm o objetivo de destacar os sinais e sintomas mais comuns da valvopatia, guiar a solicitação e a avaliação emergencial de exames complementares e discutir o diagnóstico e o tratamento das principais emergências cardiovasculares.
