# Supplementary material for: Emergências Relacionadas à Doença Valvar Cardíaca: Uma Revisão Abrangente da Abordagem Inicial no Departamento de Emergência
Source: Arq Bras Cardiol. 2023 Jun 7;120(5):e20220707. [Article in Portuguese] doi: 10.36660/abc.20220707 (PMC12092064; doi:10.36660/abc.20220707)
Supplement: Translation

# Translation: Valvular Heart Disease Emergencies: A Comprehensive Review Focusing on the Initial Approach in the Emergency Department

**Authors:** Tarso Augusto Duenhas Accorsi, Milena Ribeiro Paixão, José Leão de Souza, Marcus Vinicius Burato Gaz, Ricardo Galesso Cardoso, Karen Francine Köhler, Karine De Amicis Lima, Flavio Tarasoutchi, INTEGRATE Investigadores

**Affiliations:** 1 Brazil Unidade de Pronto Atendimento, Hospital Israelita Albert Einstein, São Paulo, SP – Brazil; 2 Brazil Instituto do Coração (InCor), Faculdade de Medicina, USP, São Paulo, SP – Brazil

**Keywords:** Valvular heart disease, heart valves, heart valve prosthesis, emergency medicine

## Abstract

Valvular heart disease (VHD) is an increasing health problem worldwide. Patients with VHD may experience several cardiovascular-related emergencies. The management of these patients is a challenge in the emergency department, especially when the previous heart condition is unknown. Specific recommendations for the initial management are currently poor. This integrative review proposes an evidence-based three-step approach from bedside VHD suspicion to the initial treatment of the emergencies. The first step is the suspicion of underlying valvular condition based on signs and symptoms. The second step comprises the attempt to confirm the diagnosis and assessment of VHD severity with complementary tests. Finally, the third step addresses the diagnosis and treatment options for heart failure, atrial fibrillation, valvular thrombosis, acute rheumatic fever, and infective endocarditis. In addition, several images of complementary tests and summary tables are provided for physician support.

## Introduction

Valvular heart disease (VHD) affects 2.5% of the population worldwide, with a marked increase after 65 years of age.^1^The natural course of VHD usually culminates in heart failure (HF).^1 - 4^Cardiac surgery is still the main definitive treatment, but advancements in transcatheter interventions have increased therapeutic options.^5^Although the recently updated VHD guidelines are focused on chronic patients,^2 - 4^however, there is a lack of specific recommendations regarding acute presentations.

Patients with VHD may experience several cardiovascular emergencies, such as acute HF, arrhythmias, thrombotic events, infective endocarditis (IE), and acute rheumatic fever (RF). When VHD is previously unknown, recognizing this underlying condition is even more challenging, especially by non-cardiologists.^6^Specific medication and intervention are required according to each valvular condition.^2 - 4^The objective of this review article is to provide an evidence-based, step-by-step approach from the suspicion of VHD in the Emergency Department (ED) to treatment of the most prevalent emergencies.

### Three-step approach

Three steps are suggested from initial VHD suspicion to the management of cardiovascular emergencies. The first step is recognizing the possibility of VHD in the ED, triggering the next step, consisting of a more detailed evaluation with complementary tests. Although echocardiography is the critical diagnostic imaging test, it is unlikely that it will be readily available. For this reason, it is essential to identify VHD signs using more available methods in the ED, such as electrocardiogram (ECG), chest radiography, and point-of-care ultrasound (POCUS). Red flags in these bedside tests should expedite the definitive echocardiographic diagnosis and severity assessment. And finally, the third step comprises diagnosing and administering specific initial treatment for the main VHD-related cardiovascular emergencies ( Central Figure ).

**Central Illustration:** : Valvular Heart Disease Emergencies: A Comprehensive Review Focusing on the Initial Approach in the Emergency Department

Step-by-step approach of valvular heart disease emergencies. VHD, Valvular heart disease; ED, Emergency department; ECG, Electrocardiogram; POCUS, Point-of-care ultrasound; HF, Heart failure; AF, Atrial fibrillation; RF, Rheumatic fever; IE, Infective endocarditis.

### First Step: Clinical suspicion of VHD in the ED

The hypothesis of VHD in the ED comes from the medical history, clinical setting, and recognition of signs in physical examination - especially the presence of heart murmur.^6 , 7^Cardiovascular symptoms appear in anatomically advanced stages in VHD as part of the natural history of the native or prosthetic valve disease ( Supplementary Figure 1 ). Less commonly, symptoms can occur as an acute onset of valve disease. The main signs and symptoms related to VHD are summarized in Table 1 .^2 - 4 , 6 , 7^

**Figure 1:** – Imaging findings of severe chronic valve heart disease. LVEF: left ventricular ejection fraction; SVI: stroke volume index; MR: mitral regurgitation.

**Table 1:** – Signs and symptoms of valvular heart disease

Symptoms	
Dyspnea, orthopnea, paroxysmal nocturnal dyspnea, edema Chest pain Syncope Palpitation Embolic events, such as stroke	
Signs	Valvulopathy
**Murmur:** – Holosystolic at the apex – Systolic crescendo-decrescendo at the right upper sternal border – Diastolic rumble at the apex, often with an opening snap – Diastolic decrescendo usually heard at the third left intercostal space – Holosystolic, increasing during inspiration at the left lower sternal border – Short mid-diastolic rumble heard loudest at the apex in the absence of an opening snap associated to holosystolic murmur	Mitral regurgitation Aortic stenosis Mitral stenosis Aortic regurgitation Tricuspid regurgitation Mitral valvulitis in acute RF (Carey Coombs)
**Pulse:** – Bounding pulse (Watson’s water hammer or Corrigan’s) – Late weak pulse ( *parvus et tardus* )	Aortic regurgitation Aortic stenosis
**Head:** – Rosy-purple cheeks (mitral facies) – Bobbing head with each heartbeat (de Musset) – Visible pulsation of the uvula (Muller) – Visible pulsation of pupils (Landolfi)	Mitral stenosis Aortic regurgitation
**Eyes:** – White centered retinal hemorrhage (Roth spots)	Infective endocarditis
**Neck:** – Giant systolic pulsations with prominent V-waves (Lancisi) – Dancing carotids (Corrigan)	Tricuspid regurgitation Aortic regurgitation
**Fingers:** – Capillary pulsation with light compression of the nail (Quincke) – Digital clubbing – Splinter lesions	Aortic regurgitation Congenital heart diseases, infective endocarditis Infective endocarditis
**Abdomen:** – Pulsation of the spleen (Gerhardt) and liver (Rosenbach)	Aortic regurgitation
**Skin:** – Subcutaneous nodules – Erythema marginatum – Osler nodules – Janeway lesions – Petechiae	Acute rheumatic fever Infective endocarditis

RF: Rheumatic fever.

The ED environment limits the ideal assessment of medical history and physical examination due to lack of privacy, crowding, noise, and limited time dedicated to each patient.^8^Furthermore, valve murmur, such as acute aortic regurgitation and mitral regurgitation, are often barely audible or even inaudible due to little pressure variability between the cardiac chambers. The transmission or murmurs can also be impaired by respiratory distress.^9^Therefore, emergency physicians should be aware of the possibility of VHD in cardiovascular emergencies.

### Second Step: complementary tests assessment for suspected VHD

VHD-related emergencies are mainly due to severe valve impairment, a condition associated with multiple cardiac anatomical changes. Bedside assessment of the ECG, chest radiography, POCUS, and in some cases, chest tomography can predict the diagnosis of VHD.^2 - 4^The standard tests for chronic VHD and acute VHD are described below. The main echocardiographic findings have been summarized so that the emergency physician can search for some of them using POCUS, and give extra attention to this data while evaluating the echocardiogram report.

### Chronic severe VHD

#### Severe chronic aortic stenosis

Severe aortic stenosis (AS) induces marked left ventricular (LV) concentric hypertrophy that can be detected on the ECG, chest radiography, and POCUS.^2 , 10^POCUS can also show calcific aortic valve with decreased movement.^10^The tomographic detection and quantification of aortic calcification is a valuable marker of severe AS.^11^The most important factor on the echocardiogram is the reduction of the aortic valve area ( Figure 1 ).^2 , 12^

#### Severe chronic aortic regurgitation

Large eccentric LV remodeling is the main feature of aortic regurgitation (AR), easily identified in bedside tests.^13^Echocardiographic criteria are based on quantitative measures of the regurgitant jet ( Figure 1 ).^2 , 12^

#### Severe chronic mitral stenosis

Mitral stenosis (MS) is a cause of HF without LV overload. Imaging of severe MS may show left atrial overload, pulmonary hypertension, and secondary remodeling of the right chambers.^14^The echocardiographic criteria are focused on reduced valve area and calcification pattern ( Figure 1 ).^2 , 12^

#### Severe chronic mitral regurgitation

Mitral regurgitation (MR) is one of the most prevalent VHDs. Severe MR usually involve left atrial overload and a moderate LV eccentric remodeling.^15^The echocardiographic criteria are based on quantitative measures of the regurgitant jet ( Figure 1 ).^2 , 12^In addition to assessing severity, for long-term treatment it is important to differentiate primary from secondary MR.

#### Acute severe VHD

Almost all acute VHDs involve mitral or aortic regurgitation.^9 , 13 , 15^The leading causes are IE, acute RF, procedure-related injury (i.e., percutaneous balloon valvuloplasty or cardiac catheterization), spontaneous prosthesis rupture, and blunt trauma.^16 , 17^AR can also be caused by type A aortic dissection and MR by acute coronary syndrome (leaflet tethering), chordal rupture, and acute cardiomyopathy (i.e., takotsubo syndrome, peripartum and viral cardiomyopathy).^17^Acute stenosis is rare, mainly due to prosthesis thrombosis.^17^

There are no typical findings of acute VHD in the ECG. Pulmonary congestion is often seen in chest radiography and in POCUS. Although an asymmetric pulmonary edema in the right upper lung may be caused by acute mitral regurgitation, even this congestion pattern is insufficient to define the valvulopathy.^16^Thus, echocardiogram - and eventually a POCUS performed by a trained professional - is the most accurate strategy to diagnose acute VHD.^16 - 18^

## Complementary assessment

Brain natriuretic peptide (BNP) is an accurate prognostic biomarker in patients with heart diseases, such as HF. However, plasma levels of BNP are often within the normal range in patients with VHD despite cardiac remodeling and HF.^19^Higher BNP levels in patients with aortic stenosis, aortic regurgitation and MR are associated with increased left atrial size and pulmonary pressure, reduced exercise capacity, and poorer prognosis.^4 , 19^Consequently, BNP concentrations are unreliable for identifying severe VHD, but increased plasma levels may suggest worse physical performance and outcomes.

VHD patients present reduced left ventricular ejection fraction (LVEF) in advanced chronic conditions, mainly in mitral and aortic regurgitation. POCUS is useful to assess LV systolic function. The subjective impression of impaired LV contraction ( Supplementary Figure 2 ) has a significant correlation with LVEF on echocardiography. In the ED of a Brazilian cardiology center, patients with HF secondary to VHD had MR in 27.5% of the cases, aortic stenosis in 23%, aortic regurgitation in 13%, and MS in 11%.^20^

Pulmonary auscultation may be normal in VHD patients, even with significant pulmonary congestion. In this scenario, lung POCUS has a high positive likelihood ratio for HF diagnosis when at least three B-lines are identified in a longitudinal plane between two ribs in two or more regions bilaterally ( Supplementary Figure 3 ).^21^

## Third step: diagnosis and treatment of VHD-related emergencies

The treatment of patients with VHD-related cardiovascular emergencies has several particularities. The main approach for diagnosing and treating HF, atrial fibrillation (AF), valvular thrombosis, RF, and IE is discussed below.

For this step, the emergency physician must ensure that the patient and other specialists participate in the decision-making process. Multidisciplinary discussion should be encouraged in all centers, especially for critical therapeutic decisions. Clinical cardiologists, echocardiographers, interventional cardiologists, cardiac surgeons, infectious disease physicians, anesthesiologists and radiologists are often part of the Heart Team. In more complex cases, other professionals may be required.^2 - 4^

### Heart failure

Progressive HF in chronic VHD patients is the leading cause of emergency care.^22^The typical findings of HF due to VHD are the same as the other causes: dyspnea, orthopnea, tachycardia, abnormal apical impulse, low systolic blood pressure, third heart sound, jugular venous distention, and edema. In sudden onset or rapid progression of pulmonary edema and hemodynamic instability, acute VHD is more common.^16 , 17^Even in non-hypotensive patients, cardiogenic shock should be considered in patients presenting with fatigue, weakness, dizziness, decreased level of consciousness, syncope, increased heart rate, increased respiratory rate, livedo, and history of diminished diuresis.^23 - 25^

VHD has different hemodynamic mechanisms. Thus, management is individualized based on the pathophysiology of each valvular condition.^2^LV work can be represented by a pressure and volume (PV) curve ( Supplementary Figure 4 ).^26^A normal LV has low-pressure cycles and good compliance.^27^As the cardiac output depends on the preload, afterload, and inotropism, changes in these parameters affect the PV curve. Both chronic and acute VHD modify the PV curve. For example, 1) due to right deviation of the LV volume curve in MS, there is little benefit of measures to increase inotropism in the presence of low output; 2) in aortic stenosis, there is high LV pressure; therefore, a substantial reduction in LV volume with diuretic therapy can induce low output; and 3) Vasodilation in regurgitant lesions is essential to reduce ventricular filling pressures and relieve congestion.^28^The purpose of drug treatment is to readjust these parameters until definitive invasive valve treatment. Therefore, specific medications and interventions are required according to each valvulopathy, often different from those used in other HF etiologies.

For severe aortic stenosis, due to a fixed cardiac output, diuretics are the mainstay of treatment. Vasodilatation and beta blockers should be avoided due to the possibility of decreasing cardiac output. The management of cardiogenic shock includes some precautions: avoid tachycardia caused by vasoactive drugs, avoid fluid therapy as most patients are hypervolemic, and consider the use of intra-aortic balloon pump (IABP), extracorporeal membrane oxygenation, and percutaneous balloon aortic valvuloplasty as temporary strategies for hemodynamic control as a bridge to definitive intervention.^2 , 24 , 25 , 29 , 30^

Aortic and mitral regurgitation benefit from vasodilation and diuretics, usually with good clinical response.^2 , 31 - 33^Beta-blockers and calcium channel blockers are not part of standard therapy and are intended to control heart rate in patients with AF with a high ventricular response.^2^IABP is contraindicated in aortic regurgitation because it accentuates valve dysfunction and decreases cardiac output. There is evidence that IABP can be beneficial in MR, for example, as a bridging support for patients with papillary rupture after myocardial infarction until surgery.^24 , 34^

MS is the only valvulopathy in which beta-blockers and calcium channel blockers are part of the main therapy.^2 , 35 - 37^Ivabradine can be used in sinus rhythm as an alternative for patients intolerant to beta-blockers or combined to beta-blockers if heart rate remains above 60 bpm.^36^Digoxin is the option for patients with AF. Diuretics can also be helpful for congestion management.^2 , 35 - 38^In flash pulmonary edema, first-line therapy includes heart rate control and diuretics. In this case, if the patient has confirmed or presumed right ventricular dysfunction (signs and symptoms of right HF), digitalis are preferred over beta-blockers to maintain myocardial contractility, and invasive and non-invasive ventilation should be avoided as increased thoracic pressure results in decreased right ventricular preload.^36^During pregnancy, the physiological increase in blood volume and heart rate imposes a greater risk of decompensation, even in previously asymptomatic women. For this population, the main pharmacological options are propranolol or metoprolol, and digoxin. Percutaneous balloon mitral valvuloplasty can be performed during pregnancy and for patients who are refractory to drug therapy if the anatomy is favorable.^24 , 39^

Detailed indications of fluid therapy, vasopressors, IABP, other bridge interventionist strategies, non-invasive ventilation, advanced airway management, diuretics, and optimization of the LV PV curve are detailed in Table 2 .^2 - 4 , 23 - 25 , 29 , 31 - 51^The most frequently used medication doses are described in Supplementary Table 1 .^2 - 4 , 23 - 25 , 29 , 31 - 33 , 35 - 37 , 47 - 50^

**Table 2:** – Hemodynamic management in the emergency department

Management of organic perfusion in hypotensive patients				
	Aortic stenosis	Aortic regurgitation	Mitral stenosis	Mitral regurgitation
Fluid therapy: Crystalloid a) Isotonic b) Avoid hypertonic and hypotonic solutions No strong evidence for colloid volume expanders.	Attention: most patients are congested. Usually, fluid therapy is not appropriate and may induce respiratory distress. Fluids should be administered with unequivocal clinical and ultrasonographic signs of hypovolemia.			
	Normal LVEF + “ *kissing walls* ” + IVC < 10mm + absence of B-lines Highly preload dependent	Normal LVEF + IVC < 10mm + absence of B-lines	HR < 100 bpm (usually on medication) + RV diameter < LV diameter + absence of D-shape + IVC < 10 mm + absence of B-lines	LVEF + RV diameter < LV diameter + absence of D-shape + IVC < 10 mm + absence of B-lines
Vasopressors	Low dose dobutamine – avoid tachycardia.	Dobutamine	Avoid dobutamine (avoid tachycardia)	Dobutamine
	Low dose norepinephrine – avoid tachycardia	Norepinephrine	Low dose norepinephrine – avoid tachycardia.	Norepinephrine
Other drugs	Short-acting vasoactive amines are options (phenylephrine)	No strong supportive evidence	If tachycardia (even sinus rhythm), beta blocker may be used; short-acting vasoactive amines may be options (phenylephrine). Milrinone if PAH.	Milrinone if PAH
Intra-aortic balloon	Possible benefit	Contraindicated	No evidence	Possible benefit
Interventionist / other strategies	Consider the percutaneous balloon valvuloplasty as a bridge for definitive intervention; extracorporeal membrane oxygenation as a bridge for definitive intervention	No strong supportive evidencea	Consider percutaneous balloon mitral valvuloplasty if favorable (anatomical criteria and absence of contraindications)	Percutaneous ventricular assist device as a bridge for definitive intervention. Without current evidence for MitraClip
**Management of hypoxemia**				
	**Aortic stenosis**	**Aortic regurgitation**	**Mitral stenosis**	**Mitral regurgitation**
Non-invasive ventilation: avoid deep sedation and opioids	Possible, even in mild hypotension	Possible, even in mild hypotension	Possible, even in mild hypotension; avoid in severe PAH and/or RV dysfunction	Possible, even in mild hypotension; avoid in severe PAH and/or RV dysfunction
Advanced airway management No strong evidence for premedication with lidocaine Sedation strategies (choose one): a) Propofol b) Etomidate c) Ketamine d) Midazolam plus (choose one): a) Succinylcholine b) Rocuronium Initial mechanical ventilation: tidal volume 6 mL/Kg, plateau pressure < 30 mmHg, titrated PEEP and driving pressure < 20 mmHg	Hypotension often occurs after intubation; attention to the choice of medications during sedation and maintain vasopressors readily available	Intubation is usually well tolerated	Avoid ketamine; marked hypotension after intubation when there is PAH	Intubation is usually well tolerated
Diuretics (furosemide; no evidence for other classes in emergency setting)	Administer only if lung congestion; avoid if compensated oxygenation	Usually necessary	Usually necessary	Usually necessary
**ptimize the pressure-volume curve until definitive treatment**				
	**Aortic stenosis**	**Aortic regurgitation**	**Mitral stenosis**	**Mitral regurgitation**
Rhythm Amiodarone can be used in all scenarios according to clinical judgment in stable patients with acute supraventricular arrhythmia.	Maintain sinus rhythm if possible	Consider chronic stable AF as sinus tachycardia. Diltiazem and esmolol may be carefully used.	Maintain sinus rhythm if possible	Consider chronic stable AF as sinus tachycardia. Diltiazem and esmolol may be carefully used.
Heart rate	Avoid excessive tachycardia in non-sinus rhythm with amiodarone, diltiazem, verapamil, esmolol, metoprolol tartrate, lanatoside C	Avoid routine use of beta blockers. Diltiazem and esmolol may be carefully used.	Avoid tachycardia in all rhythms with amiodarone, diltiazem, verapamil, esmolol, metoprolol tartrate, lanatoside C	80–100 bpm Avoid intense decrease. Diltiazem and esmolol may be carefully used
Preload: POCUS should monitor IVC and other dynamic parameters	Avoid diuretics and nitrates (nitroglycerin, isosorbide)	In stable patients with lung congestion, it is reasonable to use of vasodilator regardless of class -nicardipin, hydralazine, captopril, and enalapril	Avoid routine use of vasodilators	In stable patients with lung congestion, it is reasonable to use of vasodilator regardless of class -nicardipin, hydralazine, captopril, and enalapril
Afterload	Nitroprusside if MAP > 60 mmHg, especially if low LVEF; avoid fast SV reduction (worsening of coronary perfusion)	Nitroprusside. Nitroglycerin should be an alternative. In stable patients with lung congestion, it is reasonable to use of vasodilator regardless of class -nicardipin, hydralazine, captopril, and enalapril	Avoid low afterload (decreases coronary perfusion)	Prevent increase; nitroprusside; nitroglycerin should be an alternative. In stable patients with lung congestion is reasonable to use of vasodilator regardless of class -nicardipin, hydralazine, captopril, and enalapril
Contractility	Levosimendan	No strong supportive evidence	No strong supportive evidence	Avoid myocardial depression

LVEF: left ventricular ejection fraction; IVC: inferior vena cava; HR: heart rate; RV: right ventricle; MAP: mean arterial pressure; SV: stroke volume; PAH: pulmonary arterial hypertension; RV: right ventricular; LV: left ventricular

Clinical and interventional cardiologists should be part of the team to discuss the best practice in these cases. In the absence of symptom control with drug treatment in non-specialized centers, patients should be promptly referred to a cardiology center for specialized treatments such as IABP, balloon valvuloplasty and cardiac surgery.

### Atrial fibrillation

VHD may present with arrhythmias, predominantly AF. The possibility of VHD should be considered in every AF scenario in the ED, especially pertaining to hemodynamic instability.^52^More than 30% of patients with AF have VHD.^53^

AF cardioversion in the ED is performed exclusively whether patient instability is due to arrhythmia. In most cases, the safest approach is heart rate control and start anticoagulation if indicated. In order to avoid cardioversion-induced stroke, the procedure is recommended after excluding atrial thrombus by echocardiogram or after three weeks of proper anticoagulation.^54^

In the long-term, there is benefit in maintaining sinus rhythm whenever possible. The atrial size is not used to contraindicate cardioversion; however, the more significant the remodeling, the less chance of AF reversal and maintenance. Other recurrence risk factors in AF include years, age, renal dysfunction, and other cardiovascular risk factors.^4 , 54^

Stroke is the most undesirable event in patients with AF.^54 , 55^VHD accounts for nearly one-third of all ischemic strokes between 15 and 45 years of age.^56^For this reason, the assessment of potential risks and benefits of anticoagulation is recommended for VHD patients with AF. Vitamin K antagonists (VKA) are the option for mechanical prosthesis (the RE-ALIGN trial^57^was prematurely interrupted due to events in the dabigatran group) and MS (INVICTUS trial).^58^Non-VKA oral anticoagulants (NOAC) are recommended in other VHD, including biological prostheses (RIVER trial).^2 - 4 , 59^The PROACT Xa study, which compared the use of apixaban with warfarin for On-X mechanical aortic valve replacement, was discontinued due to a higher incidence of events in the apixaban group.^60^After the new onset of AF, the initial control of anticoagulation can be conducted on an outpatient basis, even for VKA patients. For patients at high thrombotic risk and low risk of bleeding, enoxaparin use can be considered in VKA patients until reaching the therapeutic goal. Bridging is not applicable for NOAC patients in any setting.^61^

### Valvular thrombosis

Prosthesis thrombosis is characterized by a thrombus formation on the prosthetic structures, with subsequent valve dysfunction with or without thromboembolism.^62^This is more common in the mechanical prostheses, ranging from 0.1% to 5.7%, especially in the early perioperative period, mitral position, and subtherapeutic anticoagulation.^63^

Prosthesis thrombosis can manifest in different ways, depending on valve dysfunction severity, and on thrombus size and mobility. Patients may be asymptomatic with incidentally detected thrombus and, in other cases, it can cause embolism, hypotension, syncope, dyspnea, pulmonary congestion, and sudden death.^64 , 65^Diagnosis is classically confirmed by transesophageal echocardiogram, but a multidetector computed tomography and radioscopy evaluation may also be useful.

Non-obstructive prosthesis thrombosis in stable patients is treated with optimization of oral anticoagulation. Fibrinolysis or surgery is recommended for patients with remaining thrombus after optimal anticoagulation or with thrombus ≥10mm and/or >0,8cm^2^associated with emboli. Hemodynamically unstable patients should promptly be submitted to valve replacement. For patients deemed to be at a prohibitive risk for surgery, fibrinolysis is the main option. Fibrinolysis is performed with alteplase 90 mg in 90 minutes or streptokinase 1,500,000 UI in 60 minutes. Potential complications of this treatment are bleeding, embolism, and recurrence of thrombosis.^2 - 4 , 66^These are high risk patients, therefore, the decision-making process should involve the clinical cardiologist and cardiac surgeon.

### Rheumatic fever

RF is an autoimmune response to group A Streptococcus pharyngeal infection which occurs two to four weeks after the exposure. Genetically susceptible individuals account for 0.1 to 5% of the population.^67 - 69^The first episode usually manifests in school-age children in low-income regions.^67 - 70^Environmental factors related to high levels of streptococcal infection are household overcrowding, poor sanitation, and lower use of antibiotics for pharyngitis.^71^The clinical presentation is summarized in Table 3 .^67 , 68^There is no diagnostic laboratory test for RF; the diagnosis should meet the revised Jones criteria ( Table 4 ).^67 , 68 , 72^

**Table 3:** – Clinical manifestations of acute rheumatic fever

Manifestation	Prevalence (%)	Signs and symptoms
Carditis	50–70	Valvulitis is the main manifestation, although myocarditis and pericarditis can also occur; if valvulitis is severe, acute heart failure can occur; subclinical carditis refers to echocardiographic diagnosis in the absence of auscultatory findings.
Arthritis	35–66	Migratory polyarthritis, remaining 1 to 7 days in each joint; larger joints are chiefly affected: knees, ankles, elbows, and wrists; pain is more intense than the clinical findings; absence of long-term deformity; early onset of NSAID can induce monoarthritis, due to its rapid improvement.
Sydenham chorea	10–30	Involuntary, abrupt, nonrhythmic movements of trunk, extremities, head, face, and tongue; related to emotional lability and muscle weakness; disappears during sleep; usually starts three months after GAS infection and lasts 2 to 3 months.
Subcutaneous nodules	0–10	Firm, painless, up to 2 centimeters, often 3 to 4 nodules, located over a bone or extensor surface of tendons (commonly on the olecranon), with normal skin surrounding; appears 1 to 2 weeks after other manifestations and lasts less than a month; related to carditis; almost never occurs as the only RF manifestation.
Erythema *marginatum*	< 6	Pink rash with pale center and rounded or with serpiginous borders on the trunk or proximal extremities (face preserved); lesions blanches with pressure and can appear, disappear, and reappear in hours; related to carditis; almost never occurs as the only RF manifestation.

NSAID: non-steroidal anti-inflammatory drugs; RF: rheumatic fever.

**Table 4:** – Current diagnostic criteria for rheumatic fever

	High risk groups (living in an endemic setting, personal history and age < 40 years, prior residence or frequent or recent travel in a setting of high risk for rheumatic disease)	Low risk groups (no high-risk conditions)
Major manifestations	carditis (including subclinical evidence of valvulitis on echocardiogram) polyarthritis or aseptic monoarthritis or polyarthralgia Sydenham chorea erythema *marginatum* subcutaneous nodules	carditis (including subclinical evidence of valvulitis on echocardiogram) polyarthritis Sydenham chorea erythema *marginatum* subcutaneous nodules
Minor manifestations	fever > 38ºC monoarthralgia erythrocyte sedimentation rate > 30 mm/h or C-reactive protein > 30 mg/L d) prolonged PR interval on ECG	fever > 38.5ºC polyarthralgia or aseptic monoarthritis erythrocyte sedimentation rate > 60 mm/h or C-reactive protein > 30 mg/L dprolonged PR interval on ECG
Definite initial episode of acute RF	2 major manifestations + evidence of preceding GAS infection; OR 1 major + 2 minor manifestations + evidence of preceding GAS infection.	
Definite recurrent episode of acute RF in patients with documented history of acute RF or RHD	2 major manifestations + evidence of preceding GAS infection; OR 1 major + 2 minor manifestations + evidence of preceding GAS infection; OR 3 minor manifestations + evidence of preceding GAS infection.	
Probable or possible acute RF (initial or recurrence)	1 major or 1 minor manifestations; OR no evidence of preceding GAS infection. Probable acute RF rather than highly suspect Possible acute RF rather than uncertain	

RF: rheumatic fever; GAS: group A streptococcal; RHD: rheumatic heart disease; ECG: electrocardiogram.

Treatment of acute RF has three pillars in addition to those mentioned for VHD: eradication of the inciting group A streptococcal infection, drug management according to manifestations, and prophylaxis ( Table 5 ).^67 , 68 , 73^

**Table 5:** – Rheumatic fever treatment

	Treatment
All cases	Eradication of the GAS infection: – Benzathine benzylpenicillin G 1,200,000 units (child < 20 kg: 600,000 units; ≥ 20 kg: 1,200,000 units) intramuscular single dose – Penicillin hypersensitivity: cephalexin 1 g (child: 25 mg/kg up to 1 g) orally, 12-hourly for 10 days or azithromycin 500 mg (child: 12 mg/kg up to 500 mg) orally daily for 5 days
Carditis	Rest, with mobilization as symptoms allow Prednisone/prednisolone 1–2 mg/kg up to a maximum of 80 mg orally, once daily or in divided doses for 4 to 8 weeks in mild carditis and 12 weeks in moderate/severe carditis. After 2 to 3 weeks, if symptoms improve, reduce the dose by 20 to 25% weekly. In refractory cases, 30 mg/kg/day weekly. Pediatric dosing: furosemide 1–2 mg/kg orally as a single dose, then 0.5–1 mg/kg (to a maximum of 6 mg/kg) orally, 6- to 24-hourly; spironolactone 1–3 mg/kg (initially) up to 100 mg orally, daily in 1 to 3 divided doses (round dose to a multiple of 6.25 mg – a quarter of a 25 mg tablet); enalapril 0.1 mg/kg orally, daily in 1 or 2 divided doses increased gradually over 2 weeks to a maximum of 1 mg/kg orally, daily in 1 or 2 divided doses, other ACE inhibitors (captopril, lisinopril, ramipril, perindopril) can be used. Adult dosing: Furosemide 20–40 mg oral or intravenous as a single dose followed by 20–40 mg oral or intravenous 8–12 hourly; spironolactone may be added for patients having limited or no response to loop diuretic, 12.5–25 mg spironolactone orally daily; nitrate therapy may be added for patients with limited or no response to diuretic therapy, whose systolic blood pressure is greater than 90 mmHg; the ACE inhibitor is recommended in patients with moderate or severe left ventricular systolic dysfunction, unless contraindicated. Valve surgery for life-threatening acute carditis (rare)
Arthritis	Lasts about one month without treatment Rapid improvement with NSAID, often 1 to 2 weeks (may be up to 12 weeks) – Naproxen immediate-release 250–500 mg (child 10–20 mg/kg/day) orally twice daily, up to a maximum of 1250 mg daily – Ibuprofen 200–400 mg (child 5-10 mg/kg) orally thrice daily, up to a maximum of 2400 mg daily – Aspirin adults and children 50–60 mg/kg/day orally, in 4 to 5 divided doses; dose can be escalated up to a maximum of 80–100 mg/kg/day in 4 to 5 divided doses Proton pump inhibitor should be considered. Corticosteroids are used if intolerance or allergy to NSAID or if indicated to treat another manifestation, i.e., carditis.
Chorea	No pharmacological treatment for mild cases; for moderate/severe cases: – Haloperidol 0.5 mg per dose orally, twice daily (can be increased 0.5 mg/day until 5mg/day) – Carbamazepine 3.5–10 mg/kg per dose orally, twice daily – Sodium valproate 7.5–10 mg/kg per dose orally, twice daily Consider adding corticosteroid: prednisone/prednisolone 1–2 mg/kg up to a maximum of 80 mg orally, once daily or in divided doses
Prophylaxis	Penicillin G benzathine intramuscular – Adults > 20 kg: 1.2 million units every 21 to 28 days – Children ≤ 20 kg: 600,000 units every 21 to 28 days If penicillin allergy: – Sulfadiazine: < 30 kg 500 mg orally, once daily; ≤ 30 kg: 1 g orally, once daily – Erythromycin: 250 mg orally, twice daily Rheumatic fever without carditis: 5 years or until 21 years of age (whichever is longer) Rheumatic fever with carditis but no residual heart disease (no clinical or echocardiographic evidence of valvular disease): 10 years or until 21 years of age (whichever is longer) Rheumatic fever with carditis and residual heart disease (persistent valvular disease): 10 years or until 40 years of age (whichever is longer); sometimes lifelong prophylaxis (i.e., healthcare professionals, infant professors)

GAS: group A streptococcal; ACE: angiotensin-converting enzyme; NSAID: non-steroidal anti-inflammatory drugs.

### Infective endocarditis

IE is an infection of a native or prosthetic heart valve, endocardial surface, or an indwelling cardiac device. Despite the improved diagnostic and therapeutic strategies, one-year mortality remains at 30%.^74^

The diagnosis should be considered in patients without clear infectious focusing on clinical evaluations, and the presence of heart murmur or a predisposing heart condition with one of the following: fever, suspicion of systemic emboli, and acute/subacute heart failure. Other symptoms and signs such as arthralgia, myalgia, anorexia, weight loss, night sweat, chills, headache, abdominal pain, back pain, dyspnea, and hematuria can also be present. The main predisposing heart conditions include pre-existing VHD, history of IE, and intravenous drug use. Some physical manifestations can raise the suspicion of IE, such as Roth spots - white-centered retinal hemorrhage, Osler nodules - painful nodes on the tips of fingers or toes, Janeway lesions - painless, nontender, erythematous, or hemorrhagic macular or nodular lesions on the palms or soles, Splinter hemorrhage - painless hemorrhages in the distal third of the nail, and petechiae - present in skin and mucous membranes.^75 - 78^

IE has a varied clinical presentation. The anatomopathological criteria are obtained only in 20 to 40% of the cases. The Duke criteria are a set of diagnostic criteria for IE, but their the main limitations are that they are not immediate and the accuracy at admission is around 52 to 70% (Supplementary Table 2 ).^75 - 78^

The two pillars for the treatment of IE are antibiotics ( Table 6 ) and surgical treatment (Supplementary Table 3 ).^75 - 80^In the ED, antibiotics are provided empirically based on the type of valve and IE.^75 , 76^The primary goals of surgery include valve exploration, debridement, and reconstruction or replacement of the valve/prosthesis.^79^In general, delaying surgery until completion of antibiotic therapy is associated with a higher frequency of the combined outcome of death, embolic events, and recurrence of IE in the first years after initial infection. Therefore, surgical treatment should be considered since the beginning of treatment and constantly evaluated.^75 - 80^

**Table 6:** – Empirical antibiotic regimen for infective endocarditis in emergency department

Type of infective endocarditis	Antibiotic regimen and dose	
Native valve endocarditis or prosthetic valve (> 1 year of surgery)	Ampicillin 2 g IV every 4 hours + Oxacillin 2 g IV every 4 hours + Gentamicin* 1 to 3 mg/Kg of current body weight IV every 24 h or divided in 3 doses Alternative in cases of low probability of enterococci: ceftriaxone 1g every 12 hours instead of ampicillin + oxacillin 2g IV every 4 hours	4 to 6 weeks** 4 to 6 weeks** 2 weeks 4 to 6 weeks
Prosthetic valve (< 1 year of surgery)	Vancomycin 15 mg/Kg IV every 12 hours or Daptomycin 8 to 10 mg/Kg IV every 24 hours Meropenem 2 g IV every 8 hours or Cefepime 2 g IV every 8 hours + Gentamicin* 1 to 3 mg/Kg of actual body weight IV every 24 h or divided in 3 doses	6 weeks 6 weeks 2 weeks

IV: intravenously; * except in chronic kidney disease or occurrence of nephrotoxicity; **4 weeks for native and 6 weeks for prosthesis.

Multiple professionals should be part of the patient’s care. In addition to the emergency physician, the nursing staff, infectious disease physicians, clinical cardiologists and cardiac surgeons, the Endocarditis Team may also need a neurosurgeon or vascular surgeon (mycotic aneurysm, septic embolization), general or gastrointestinal surgeon (abscesses of intra-abdominal organs), orthopedist (discitis), nephrologist (toxicity of antibiotic treatment), among others.

## Conclusion

Despite the high complexity and heterogeneity of VHD-related emergencies, the three-step approach can help clinical reasoning. The steps have the objective to highlight the most common signs and symptoms of VHD, guide critical request and evaluation of complementary tests and discuss diagnosis and treatment of the main cardiovascular emergencies.
